# Case Report: Carotid body paraganglioma in a 20-year-old woman: multimodal imaging, vessel-preserving surgical excision, and long-term clinical follow-up

**DOI:** 10.3389/fsurg.2026.1837432

**Published:** 2026-07-01

**Authors:** Weixu Zhou, Dong Chen, Xianwei Wang

**Affiliations:** Department of Neurosurgery, Central Hospital Affiliated to Dalian University of Technology (Dalian Central Hospital), Dalian, China

**Keywords:** carotid body paraganglioma, carotid body tumor, case report, multimodal imaging, surgery

## Abstract

Carotid body paraganglioma is a rare hypervascular neuroendocrine tumor of the head and neck, and presentation in a 20-year-old woman is uncommon. We report a young woman with a painless left lateral neck mass present for more than 5 years, with more noticeable enlargement during the preceding year. On admission, a firm, mobile, non-tender mass was palpated along the anterior border of the left sternocleidomastoid muscle, without an audible bruit. Initial and targeted ultrasonography demonstrated a hypervascular lesion centered at the left carotid bifurcation. Preoperative contrast-enhanced computed tomography angiography (CTA) demonstrated a hypervascular mass centered at the left carotid bifurcation with widening of the bifurcation and separation of the internal and external carotid arteries; magnetic resonance imaging (MRI) showed a heterogeneously enhancing carotid-space mass with internal flow-void foci; and digital subtraction angiography (DSA) confirmed marked tumor blush, collectively supporting the diagnosis of carotid body paraganglioma. Surgical excision was performed under general anesthesia. Intraoperatively, the tumor crossed the carotid bifurcation, wrapped around the internal and external carotid arteries, and was extremely vascular; however, grossly complete, vessel-preserving excision was achieved with preservation of the carotid arteries, hypoglossal nerve, and vagus nerve. Histopathology confirmed carotid body paraganglioma with an organoid/nested architecture within a rich vascular stroma and immunohistochemistry showing synaptophysin (Syn)(+), chromogranin A (CgA)(+), S-100(+), and a Ki-67 labeling index of approximately 5%; the original pathology report did not specify a formal microscopic margin status. Balloon occlusion testing, biochemical catecholamine work-up, functional imaging, and genetic testing were not documented in the available record. The postoperative course was uneventful. Ultrasonographic follow-up on July 26, 2018 showed no definite recurrent lesion or obvious vascular abnormality, and long-term clinical follow-up by telephone revealed no recurrence-related symptoms, although no further imaging surveillance was available, and asymptomatic radiologic recurrence cannot be fully excluded. This case highlights the value of multimodal imaging in diagnosis and operative planning and shows that grossly complete, vessel-preserving excision can be associated with favorable short-term imaging and long-term symptom-based follow-up findings in a young patient.

## Introduction

Carotid body tumors are the most common paragangliomas of the head and neck and arise from paraganglionic tissue at the carotid bifurcation. They are typically slow-growing, highly vascular lesions that present as a painless lateral neck mass. Although carotid body paraganglioma is well recognized in adults, presentation in very young adults remains relatively uncommon in routine clinical practice. Multimodal imaging is central to diagnosis, anatomical localization, differential diagnosis, and operative planning (1–4), whereas surgery remains an important treatment option for resectable lesions in selected patients (5, 6). Here, we report a 20-year-old woman with left carotid body paraganglioma documented by ultrasonography, digital subtraction angiography (DSA), computed tomography angiography (CTA), magnetic resonance imaging (MRI), surgery, pathology, and both short-term imaging-based follow-up and long-term symptom-based clinical follow-up.

## Case description

A 20-year-old woman was admitted on January 18, 2018 for evaluation of a left neck mass that had been present for more than 5 years. The lesion was initially approximately peanut-sized, soft, mobile, and painless. It enlarged slowly over time and had been managed conservatively without obvious reduction. During the year before admission, the mass increased more noticeably in size.

She denied dysphagia, choking with drinking or eating, hoarseness, dizziness, headache, palpitations, and chest tightness. Appetite, sleep, and bowel habits were normal. She had no history of hypertension, diabetes mellitus, coronary artery disease, hepatitis, tuberculosis, previous surgery, transfusion, or trauma. She had a history of penicillin allergy. She did not smoke or drink alcohol. No definite hereditary disease or similar tumor history was documented in the available family history.

On admission, temperature was 36.5°C, pulse 78/min, respiratory rate 19/min, and blood pressure was 117/75 mmHg. A firm, mobile, non-tender mass measuring approximately 4 × 5 cm was palpated along the anterior border of the left sternocleidomastoid muscle. The lesion was appreciably mobile on lateral palpation. No audible bruit was present over the lesion, and no superficial lymphadenopathy was detected. Tongue protrusion showed mild leftward deviation. No ipsilateral shoulder droop or shoulder weakness was present, and limb strength was normal. Because this is a retrospective case, a formal Fontaine sign assessment was not explicitly documented in the original medical record and therefore is not overinterpreted in the present report. The main events during diagnosis, treatment, and follow-up are summarized in Table 1.

**Table 1:** Timeline of the case.

Time	Clinical event	Objective findings/significance
More than 5 years before admission	The patient noticed a left lateral neck mass	The lesion was initially approximately peanut-sized, soft, mobile, and painless.
During the year before admission	Progressive enlargement of the neck mass	Growth became more noticeable, prompting further medical evaluation.
January 17, 2018	Initial ultrasonography of the superficial neck mass	A solid hypoechoic mass was identified from the left carotid bulb to the bifurcation of the internal and external carotid arteries. The lesion measured approximately 4.5 × 3.1 × 3.0 cm and showed abundant internal vascularity, favoring carotid body tumor.
January 18, 2018	Hospital admission	A firm, mobile, non-tender mass measuring approximately 4 × 5 cm was palpated along the anterior border of the left sternocleidomastoid muscle. No bruit was heard. Mild leftward tongue deviation was noted.
January 22, 2018	Targeted neck ultrasonography	A lesion measuring approximately 4.2 × 3.4 × 2.5 cm was localized between the internal and external carotid arteries, with abundant internal and peripheral blood-flow signals.
January 22, 2018	Carotid duplex ultrasonography	No obvious hemodynamic abnormality was detected in the major cervical arteries.
January 23, 2018	Contrast-enhanced CTA	A well-defined, strongly enhancing mass centered at the left carotid bifurcation was demonstrated, with splaying of the internal and external carotid arteries.
January 24, 2018	Contrast-enhanced MRI	A left carotid-space mass with relatively clear margins, heterogeneous signal intensity, punctate flow-void foci, and marked enhancement was demonstrated, supporting carotid body origin.
January 25, 2018	Preoperative digital subtraction angiography	Angiography demonstrated a hypervascular lesion with marked tumor blush at the left carotid bifurcation, further supporting carotid body paraganglioma.
February 6, 2018	Surgical treatment	Grossly complete tumor excision was performed under general anesthesia. Intraoperatively, the tumor measured approximately 5 × 4 × 3 cm, crossed the carotid bifurcation, wrapped around the internal and external carotid arteries, and was extremely vascular. The carotid arteries, hypoglossal nerve, and vagus nerve were preserved. Estimated blood loss was 100 mL.
February 12, 2018	Pathological diagnosis	Pathology confirmed left carotid body paraganglioma. Immunohistochemistry showed synaptophysin (Syn)(+), chromogranin A (CgA)(+), S-100(+), and Ki-67 of approximately 5%, with focally increased proliferative activity and focal capsular invasion. A formal microscopic margin status was not explicitly reported.
February 15, 2018	Hospital discharge	The postoperative course was favorable, with marked improvement in neck swelling and no major cranial nerve-related symptoms documented at discharge.
July 26, 2018	Imaging follow-up	Follow-up ultrasonography showed no definite recurrent lesion at the left cervical operative site and no obvious vascular abnormality.
Subsequent years	Long-term clinical follow-up by telephone	The patient reported no recurrence-related symptoms; however, no further imaging surveillance was available after July 26, 2018, and asymptomatic radiologic recurrence cannot be fully excluded.

## Diagnostic assessment

Initial ultrasonography on January 17, 2018 demonstrated a solid hypoechoic mass extending from the left carotid bulb to the bifurcation of the internal and external carotid arteries. The lesion measured approximately 4.5 × 3.1 × 3.0 cm, lay 0.48 cm beneath the skin surface, had clear margins and a relatively regular shape, and showed abundant internal vascularity with an arterial spectral pattern, suggesting a carotid body tumor supplied by branches of the external carotid artery.

A targeted neck ultrasound on January 22, 2018 further localized the lesion between the internal and external carotid arteries. The mass measured approximately 4.2 × 3.4 × 2.5 cm, had clear margins, a slightly irregular contour, heterogeneous echogenicity with a small cystic component, and abundant internal and peripheral blood-flow signals. Carotid duplex ultrasonography performed on the same day showed no obvious hemodynamic abnormality in the main cervical arteries.

Contrast-enhanced CTA performed on January 23, 2018 demonstrated a well-defined, intensely enhancing mass centered at the left carotid bifurcation, measuring approximately 4.0 × 3.6 × 3.4 cm, with splaying of the internal and external carotid arteries. Preoperative contrast-enhanced MRI performed on January 24, 2018 demonstrated a left carotid-space mass with relatively clear margins, heterogeneous signal intensity, punctate flow-void foci, and marked post-contrast enhancement; the lesion measured approximately 4.5 × 3.4 × 3.0 cm and widened the carotid bifurcation with separation of the internal and external carotid arteries. Preoperative digital subtraction angiography performed on January 25, 2018 demonstrated a hypervascular lesion centered at the left carotid bifurcation with marked tumor blush. Routine preoperative laboratory evaluation, including blood count, serum biochemistry, coagulation profile, hepatitis screening, blood typing, and anesthetic assessment, revealed no contraindication to surgery.

**Figure 1:**
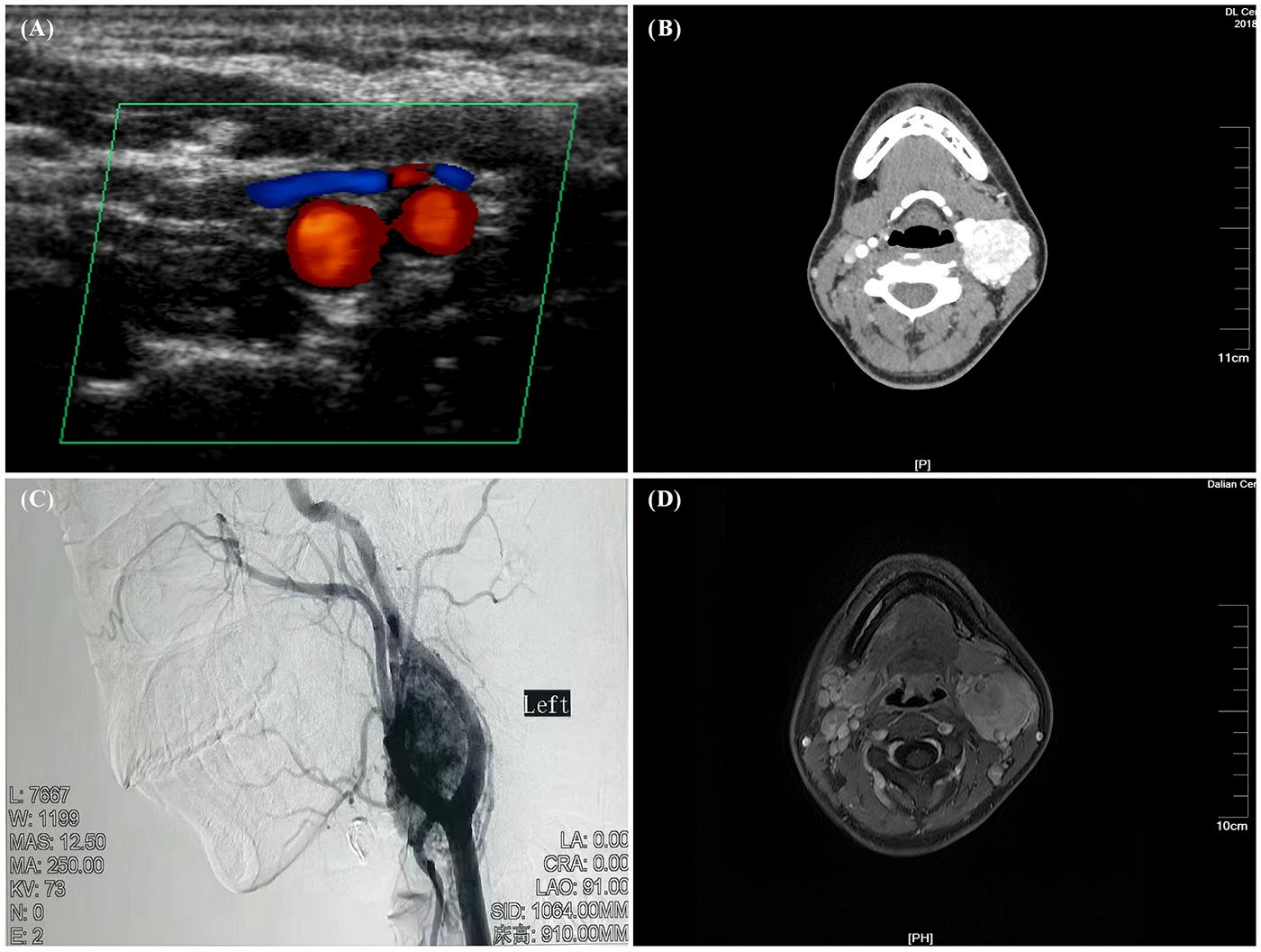
Preoperative multimodal imaging findings supporting the diagnosis and retrospective shamblin assessment of the left carotid body paraganglioma. **(A)** Preoperative color Doppler ultrasonography shows a hypervascular mass at the left carotid bifurcation. **(B)** Axial contrast-enhanced computed tomography angiography (CTA) demonstrates an intensely enhancing mass centered at the left carotid bifurcation with splaying of the internal and external carotid arteries. **(C)** Preoperative digital subtraction angiography (DSA) demonstrates marked tumor blush of a hypervascular lesion at the left carotid bifurcation. **(D)** Axial contrast-enhanced magnetic resonance imaging (MRI) demonstrates a heterogeneously enhancing carotid-space mass with internal flow-void foci. Taken together, the carotid bifurcation epicenter, splaying of the internal and external carotid arteries, hypervascular tumor blush, and close relationship to the carotid branches support carotid body origin and a retrospective assessment most consistent with Shamblin II.

Initial diagnostic considerations included carotid body tumor, vagal paraganglioma, and other neurogenic tumors. The epicenter at the carotid bifurcation, the characteristic splaying of the internal and external carotid arteries on ultrasonography, MRI, and CTA, and the marked tumor blush on DSA strongly favored carotid body origin (1–4). Although a formal preoperative Shamblin classification was not documented in the original 2018 chart, the available imaging and operative findings allow a retrospective descriptive assessment. The lesion was centered at the carotid bifurcation, widened the angle between the internal and external carotid arteries, displaced and separated these arteries, and was described intraoperatively as crossing the carotid bifurcation and wrapping around the internal and external carotid arteries. However, the carotid arteries were preserved, and neither carotid artery resection nor vascular reconstruction was required. On this basis, the lesion was considered most consistent with Shamblin II. This retrospective classification should be interpreted cautiously because it was not assigned prospectively before surgery. Balloon occlusion testing was not performed, as carotid sacrifice was not anticipated on preoperative imaging and a vessel-preserving surgical strategy was planned. The patient had no hypertension, palpitations, headache, or other catecholamine-related symptoms, and no biochemical catecholamine work-up, functional imaging, or genetic testing was documented in the available records. These absences are acknowledged as limitations of this retrospective report. Contemporary literature increasingly emphasizes hereditary evaluation and structured follow-up, particularly in younger patients (2–4, 7) (Figure 1).

## Therapeutic intervention

After multidisciplinary evaluation, the patient underwent surgical excision on February 6, 2018 under general anesthesia. A straight incision approximately 7 cm in length was made along the anterior border of the left sternocleidomastoid muscle. The carotid sheath was exposed and the common carotid artery was identified.

**Figure 2:**
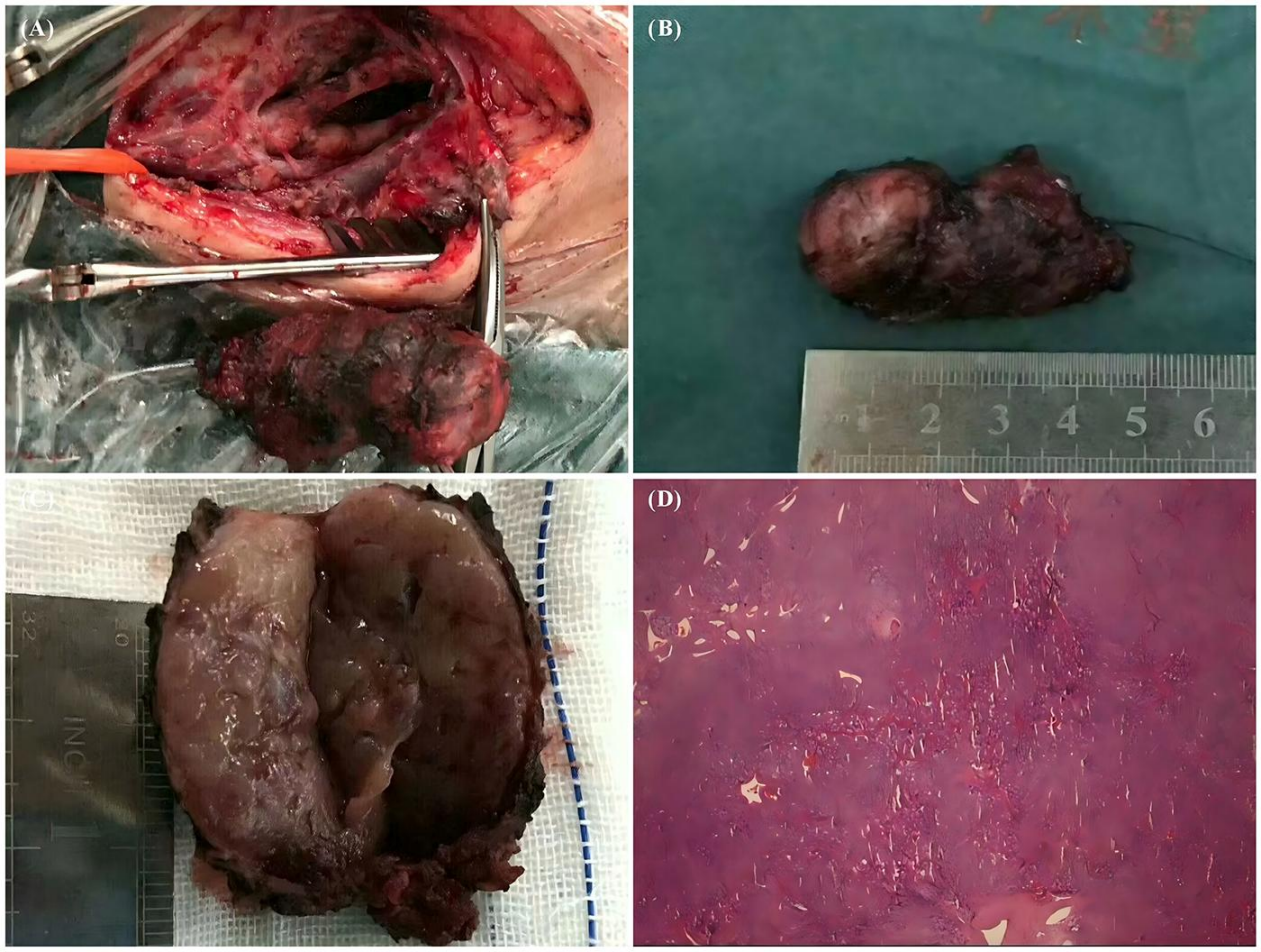
Intraoperative, gross, and histopathological findings. **(A)** Intraoperative view before tumor removal, showing the tumor occupying the left carotid bifurcation and closely related to the carotid branches. **(B)** Gross appearance of the intact resected specimen with scale. **(C)** Cut surface of the specimen, showing a solid gray-tan to gray-brown lesion. **(D)** Representative low-power histologic image from the pathology report, showing organoid/nested (zellballen-like) architecture within a vascular stroma, consistent with paraganglioma.

According to the operative record, the tumor measured approximately 5 × 4 × 3 cm, extended into the carotid sheath, crossed the carotid bifurcation, and wrapped around the internal and external carotid arteries. It was purple in color, firm in consistency, extremely vascular, and tightly adherent to the carotid arteries. Careful sharp dissection was undertaken, and the tumor was completely excised macroscopically. The internal and external carotid arteries were preserved intact without vascular reconstruction. The hypoglossal nerve and vagus nerve were identified and protected. A subcutaneous drain was placed. Estimated blood loss was 100 mL. Surgical excision remains an important treatment option for resectable carotid body tumors, particularly in young patients (5, 6, 8) (Figure 2).

## Follow-up and outcomes

The postoperative course was favorable. Neck swelling improved significantly after surgery. The patient had no fever, hoarseness, choking with drinking, shoulder droop, or shoulder weakness. She was discharged on February 15, 2018 in stable condition.

Histopathology described a gray-yellow tumor measuring approximately 4 × 2 × 2 cm. Microscopic examination was consistent with paraganglioma and showed an organoid/nested (zellballen-like) growth pattern within a rich vascular stroma. Immunohistochemistry showed synaptophysin (Syn)(+), chromogranin A (CgA)(+), and S-100(+), supporting the diagnosis of paraganglioma with neuroendocrine chief cells and sustentacular cell staining; the Ki-67 labeling index was approximately 5%. The pathology report also noted focally increased proliferative activity and focal capsular invasion. The original pathology report did not explicitly document the microscopic resection margin status. Therefore, grossly complete excision can be stated based on the operative record, but a formal R0 resection cannot be claimed from the available pathology report. These findings supported the final diagnosis of left carotid body paraganglioma. Focal capsular invasion and a Ki-67 labeling index of approximately 5% were interpreted as findings supporting continued follow-up, but not as evidence of malignancy by themselves, because malignant behavior in head and neck paraganglioma is defined by metastasis rather than by local histologic features alone.

**Figure 3:**
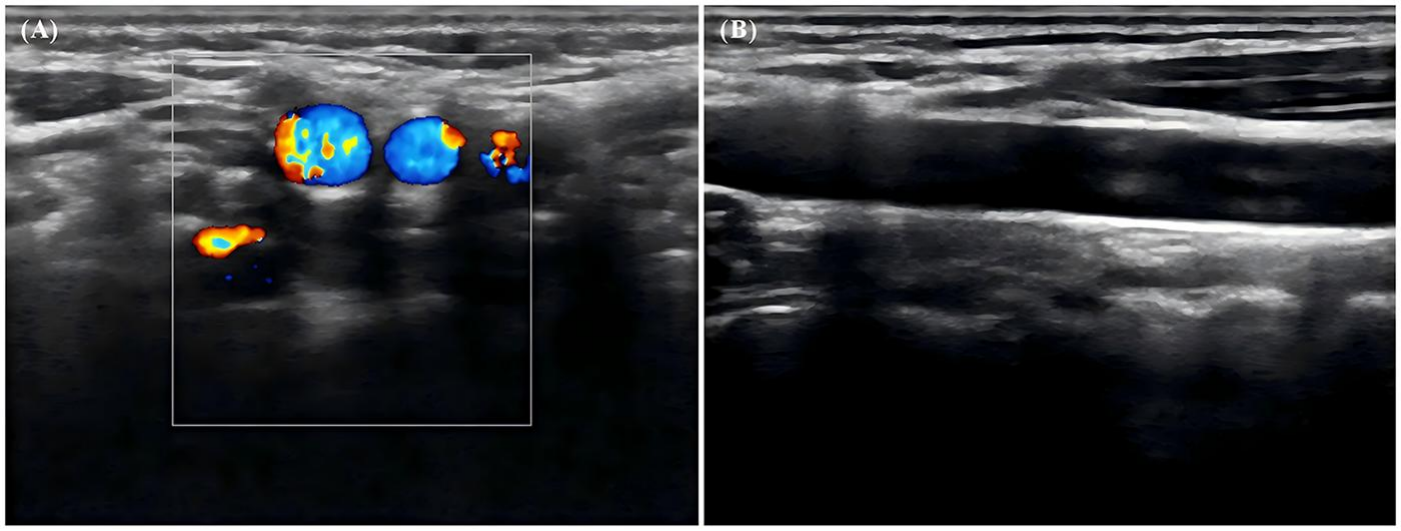
Last available postoperative imaging follow-up by ultrasonography on July 26, 2018. **(A)** Transverse color Doppler image of the postoperative left carotid bifurcation region. **(B)** Longitudinal grayscale image of the postoperative operative site. No definite recurrent mass or obvious vascular abnormality is identified on this examination; no further imaging surveillance was available thereafter.

At ultrasonographic follow-up on July 26, 2018, no definite recurrent lesion was detected at the left cervical operative site, and carotid duplex ultrasonography showed no obvious vascular abnormality. In addition, long-term clinical follow-up by telephone over the subsequent years revealed no subjective discomfort, including no recurrent neck mass, hoarseness, dysphagia, choking, or shoulder weakness. However, no further imaging surveillance was performed after the 2018 ultrasound examination; thus, the long-term follow-up after 2018 was clinical and symptom-based rather than imaging-based, and asymptomatic radiologic recurrence cannot be completely excluded (Figure 3).

## Discussion

This case is clinically instructive because of the patient's young age, the consistency of the multimodal imaging findings, and the favorable postoperative recovery after grossly complete, vessel-preserving excision. Carotid body paraganglioma is the most common head and neck paraganglioma, but presentation in a 20-year-old woman remains relatively uncommon in routine clinical practice (1, 3, 4). In a young patient with a painless lateral neck mass, a vascular paraganglioma may not be the first diagnostic consideration.

The young age of the patient is clinically relevant because hereditary paraganglioma syndromes should be considered in young patients with head and neck paraganglioma. In contemporary practice, genetic counseling and germline testing for susceptibility genes, particularly succinate dehydrogenase complex-related genes (SDHx), would be reasonable to discuss. However, this was a retrospective case from 2018, and no SDHx or other germline genetic testing was documented in the available medical record. No definite family history of similar tumors or hereditary disease was recorded. Therefore, the hereditary status of this patient remains unknown and should be recognized as an important limitation of the present report.

Compared with pediatric carotid body tumor cases summarized in a recent systematic review, in which neck mass or swelling was the most common presentation and Shamblin III lesions were frequently reported (8), the present case lies at the young-adult end of the age spectrum and is notable for concordant multimodal imaging findings, a retrospective Shamblin II assessment, grossly complete vessel-preserving excision, and favorable symptom-based long-term follow-up. However, the absence of germline genetic testing and imaging surveillance after 2018 limits direct comparison with more comprehensively evaluated contemporary cases.

In the present case, ultrasonography, DSA, CTA, and MRI established a coherent preoperative diagnosis. The most informative imaging features were the carotid bifurcation epicenter, separation of the internal and external carotid arteries, and the marked tumor blush on DSA, all of which strongly supported carotid body origin and helped distinguish this lesion from other parapharyngeal or neurogenic lesions (1–4).

On retrospective review, the carotid bifurcation epicenter, splaying and separation of the internal and external carotid arteries, close carotid-branch involvement, and successful vessel-preserving excision without vascular reconstruction were most consistent with a Shamblin II lesion, although this was not a prospectively assigned preoperative classification. This correlation between preoperative imaging and operative anatomy is one of the main educational strengths of the case. The operative findings documented a tumor that crossed the bifurcation and wrapped around the carotid branches, indicating substantial local vascular involvement. Even so, grossly complete excision was achieved with preservation of the internal and external carotid arteries, as well as the hypoglossal and vagus nerves, and blood loss was limited to 100 mL (5, 6, 8).

The pathological findings were also instructive. The organoid/nested architecture within a rich vascular stroma was morphologically typical of paraganglioma. Syn and CgA supported neuroendocrine differentiation of the chief cells, whereas S-100 highlighted the sustentacular cell component. The original pathology report did not provide a formal microscopic margin status, although the operative record described grossly complete excision. The focal capsular invasion and Ki-67 index of approximately 5% support the need for continued follow-up, but these findings should not be overinterpreted as evidence of malignancy. In head and neck paraganglioma, malignant behavior is defined by metastasis rather than by local histologic atypia alone (4, 7).

This case is not presented as a novel surgical technique; rather, its value lies in the unusually young age at presentation and the close concordance among ultrasonography, CTA, MRI, DSA, operative anatomy, and pathology. Together, these findings illustrate how multimodal evaluation can guide preoperative localization, differential diagnosis, operative planning, and retrospective surgical risk stratification in carotid body paraganglioma. Despite close involvement of the carotid bifurcation, grossly complete, vessel-preserving excision was achieved, followed by favorable postoperative recovery, no definite recurrence on short-term ultrasonographic follow-up, and no recurrence-related symptoms on long-term symptom-based telephone follow-up.

The case also highlights the limitations of a retrospective real-world dataset from 2018. A formal Fontaine sign assessment was not explicitly recorded. Balloon occlusion testing was not performed because carotid sacrifice was not anticipated and a vessel-preserving approach was planned. Biochemical catecholamine testing, functional imaging, and genetic testing were not documented in the available record. By anatomical location, the lesion was clinically classified as a carotid body paraganglioma, that is, a parasympathetic head and neck paraganglioma rather than a sympathetic paraganglioma. The absence of hypertension, palpitations, and headache was clinically consistent with a nonsecretory lesion, but biochemical confirmation was not available. Contemporary literature also increasingly emphasizes hereditary evaluation and structured follow-up, particularly in younger patients (2–4, 7). In retrospect, genetic counseling and testing would have been reasonable to discuss because of the patient's young age, even though no definite hereditary history was documented. Finally, objective imaging surveillance was available only through July 26, 2018; later follow-up was symptom-based and obtained by telephone. These limitations should be recognized when interpreting the long-term outcome. Recurrence and progression after treatment have been reported in head and neck paragangliomas, supporting the need for long-term surveillance (9, 10). Among these limitations, the absence of imaging surveillance after July 2018 is particularly important, because symptom-based telephone follow-up cannot exclude asymptomatic radiologic recurrence.

## Conclusion

In a 20-year-old woman with a slowly enlarging lateral neck mass, carotid body paraganglioma was strongly suggested by multimodal imaging and confirmed by surgery and pathology. The combination of ultrasonography, CTA, and MRI accurately demonstrated the location and carotid bifurcation anatomy of the lesion, while DSA provided additional angiographic confirmation of its marked vascularity. Grossly complete, vessel-preserving surgical excision in this patient resulted in a favorable postoperative course, no definite recurrence on short-term imaging follow-up, and no recurrence-related symptoms on long-term symptom-based telephone follow-up. At the same time, the absence of documented biochemical evaluation, functional imaging, germline genetic testing, formal microscopic margin assessment, and imaging surveillance after 2018 should be recognized as important limitations of this retrospective case.

## Patient perspective

At the latest follow-up, the patient reported no apparent discomfort after surgery and no recurrence-related symptoms.

## Data Availability

The original contributions presented in the study are included in the article/Supplementary Material, further inquiries can be directed to the corresponding author/s.
